# Induction of Human β-Defensin-2 by Vaginal *Lactobacillus crispatus* Strains in Vaginal Epithelial Cells Correlates With Their Adhesion Abilities

**DOI:** 10.1093/ofid/ofag193

**Published:** 2026-04-25

**Authors:** Masahiro Ito, Misaki Kataoka, Mio Aso, Nanae Netsu, Toshihiko Takada, Nanaka Okuda, Ayaka Sudo, Shiho Totsuka, Kirika Yoshioka, Yoichi Sato, Naoki Ogura, Hideki Nachi, Ji-hwan Pang, Nobuhiko Okada, Yun-Gi Kim, Koji Nomoto

**Affiliations:** Laboratory of Microbiology, School of Pharmacy, Kitasato University, Tokyo, Japan; Laboratory of Microbiology, School of Pharmacy, Kitasato University, Tokyo, Japan; Laboratory of Microbiology, School of Pharmacy, Kitasato University, Tokyo, Japan; Laboratory of Microbiology, School of Pharmacy, Kitasato University, Tokyo, Japan; HMS Women's Health Research & Development Center, Hanamisui Co., Ltd., Tokyo, Japan; Laboratory of Microbiology, School of Pharmacy, Kitasato University, Tokyo, Japan; Laboratory of Microbiology, School of Pharmacy, Kitasato University, Tokyo, Japan; Laboratory of Microbiology, School of Pharmacy, Kitasato University, Tokyo, Japan; Laboratory of Microbiology, School of Pharmacy, Kitasato University, Tokyo, Japan; Daikanyama Women's Clinic, Tokyo, Japan; HMS Women's Health Research & Development Center, Hanamisui Co., Ltd., Tokyo, Japan; HMS Women's Health Research & Development Center, Hanamisui Co., Ltd., Tokyo, Japan; HMS Women's Health Research & Development Center, Hanamisui Co., Ltd., Tokyo, Japan; Laboratory of Microbiology, School of Pharmacy, Kitasato University, Tokyo, Japan; Laboratory of Microbiology, School of Pharmacy, Kitasato University, Tokyo, Japan; HMS Women's Health Research & Development Center, Hanamisui Co., Ltd., Tokyo, Japan; Department of Molecular Microbiology, Faculty of Life Sciences, Tokyo University of Agriculture, Tokyo, Japan

**Keywords:** *Lactobacillus crispatus*, vaginal epithelial cell, human β-defensin-2, adhesion, bacterial viability

## Abstract

**Background:**

The human vaginal microbiota is dominated by lactobacilli, with *Lactobacillus crispatus* associated with reproductive health. Human β-defensin-2 (HβD-2), a vaginal fluid antimicrobial peptide, correlates with spontaneous births. However, their relationships remain poorly understood. We identified *L. crispatus* strains that stimulate vaginal epithelial cells to secrete HβD-2.

**Methods:**

Human vaginal epithelial cells VK2/E6E7 were co-incubated with live or heat-killed *L. crispatus* or *L. gasseri* strains. Complementary DNA was synthesized from cellular RNA, and *HβD2*, *IL8*, *TNFα*, and *MUC1* mRNA levels were quantified by quantitative PCR. HβD-2 levels were measured using ELISA. Adherent *Lactobacillus* cells on VK2/E6E7 were stained with DAPI, and bacterial cell count was determined using a fluorescence microscope.

**Results:**

Two *L. crispatus* strains, HMS-115 and HMS-122, significantly increased *HβD2* mRNA expression in VK2/E6E7 cells. The remaining eighteen strains showed *HβD2* mRNA levels comparable to those of the Dulbecco's phosphate-buffered saline control group. This increase in *HβD2* mRNA expression correlated with each strain's adhesion ability towards VK2/E6E7 cells. Heat inactivation of *L. crispatus* cells did not impair HMS-115's ability to enhance *HβD2* mRNA and protein expression, nor did it affect its adherence properties. Furthermore, incubation with live HMS-115 cells induced pro-inflammatory cytokine gene expression in VK2/E6E7 cells; however, heat-killed cells did not.

**Conclusions:**

Only *L. crispatus* strains HMS-115 and HMS-122 stimulated HβD-2 production in vaginal epithelial cells. This ability correlated with the adhesion capacity of the *Lactobacilli* to vaginal epithelial cells, regardless of bacterial viability. Thus, the beneficial effects of *L. crispatus* strains may include promoting HβD-2 production.

Lay Summary

Human β-defensin-2 (HβD-2) is a vaginal fluid antimicrobial peptide whose levels correlate with spontaneous births. In this study, we assessed *HβD2* mRNA levels in vaginal epithelial cells after incubation with various *L. crispatus* or *L. gasseri* strains. We found that the *L. crispatus* strains HMS-115 and HMS-122 stimulated HβD-2 production in vaginal epithelial cells. The ability to promote HβD-2 production in these cells correlated with strong adherence to vaginal epithelial cells. Therefore, the beneficial effects of *L. crispatus* strains may also include promoting HβD-2 production.

Preterm birth (PTB)—delivery before 37 completed weeks of gestation—is the leading cause of death among neonates and children <5 years of age [1, 2]. Globally, 1.1 million babies die annually owing to the consequences of prematurity. Approximately 15 million babies are born prematurely each year, with US annual healthcare costs for surviving infants exceeding $25 billion [3]. In Japan, data from 1979 to 2014, covering approximately 4.363 million people, show an increase in the PTB rate [4]. A primary cause of spontaneous PTB is ascending bacterial infection that originates in the vagina, moves through the cervix, and into the uterine cavity [5–7]. Therefore, maternal–vaginal microbial interactions during pregnancy substantially impact reproductive health outcomes.

In healthy premenopausal women, the vaginal mucosa is primarily colonized by one or more *Lactobacillus* species, including *L. crispatus*, *L. iners*, *L. gasseri*, and *L. jensenii* [8]. Lactobacilli produce lactic acid as a metabolite [9], which lowers vaginal pH and reduces infection risks from pathogens causing bacterial vaginosis (BV) [10] and Human Immunodeficiency Virus-1 (HIV-1) [11]. This microbial composition correlates with high rates of both spontaneous [12] and *in vitro* post-fertilization births [13, 14]. Among these species, *L. crispatus*, *L. gasseri*, and *L. jensenii* are not associated with BV [15], and *L. crispatus*, the most common vaginal microbiota among Japanese women [16], is less likely to transition than *L. iners* [17]. Furthermore, women with *L. crispatus* as their dominant vaginal species show considerably lower incidence of PTB and cervical cancer [12, 18, 19], suggesting that lactobacilli, particularly *L. crispatus*, associate with spontaneous births.

Defensins are the primary human antimicrobial peptides, playing crucial roles in host defense. Specifically, β-defensins (βD) are expressed in mucosal epithelium and skin, which are in direct contact with the external environment [20]. Human βD-2 (HβD-2), a βD peptide produced by host cells, exhibits strong antibacterial activity against Gram-negative bacteria, such as *Escherichia coli* and *Pseudomonas aeruginosa*, as well as fungi, including *Candida albicans* [21]. Although HβD-2 exhibits limited antibacterial activity against Gram-positive bacteria, including lactobacilli, it enhances bactericidal activity against *Staphylococcus aureus* in biofilms via synergistic interaction with the serine protease Esp secreted by *Staphylococcus epidermidis*, a novel mechanism distinct from all known bacterial interference mechanisms [22]. Additionally, HβD-2 exhibits antiviral activity against HIV-1 [23], respiratory syncytial virus [24], and varicella-zoster virus [25]. HβD-2 also regulates host immune responses by enhancing cytokine and chemokine expression [26]. Recently, a cohort study of 2000 pregnant women reported that elevated vaginal HβD-2 levels are associated with a reduced risk of PTB [27]. Notably, low HβD-2 levels were associated with increased PTB risk, even in women with *Lactobacillus*-dominated cervicovaginal microbiota [27]. These findings suggest that maintaining high levels of HβD-2 in vaginal fluid is more crucial for preventing PTB than *Lactobacillus* dominance alone.

*Lactobacillus* strains can induce *βD* expression in host epithelial cells [28, 29]. Additionally, surface-layer proteins (SLPs) of *L. helveticus* were reported to enhance HβD-2 production in colorectal epithelial cells [30]. Although many reports have indicated a correlation between *L. crispatus* and vaginal health, the underlying mechanisms, including the association between *L. crispatus* and HβD-2 production, remain unclear. Here, we assessed *HβD2* mRNA levels in vaginal epithelial cells after incubation with various *L. crispatus* strains isolated from healthy Japanese women [31]. We also examined the relationship between the ability of *L. crispatus* strains to enhance HβD-2 production and their adhesion to vaginal epithelial cells. We found that some vaginal *L. crispatus* strains strongly enhance HβD-2 production, and that its presence in vaginal mucus is well-correlated with spontaneous births.

## METHODS

### Bacterial Strains and Culture Conditions

*Lactobacillus crispatus* MV-1A-US was obtained from BEI Resources (Manassas, VA, USA); the other strains were from our previous study [31], which was approved by the Research Ethics Committee of Kitasato Institute Hospital (Study No. 22024) and conducted in accordance with the principles of the Declaration of Helsinki. *L. crispatus* HMS-115 and HMS-122 used in the experiment were deposited internationally at the National Product and Technology Evaluation Center (NITE) Patent Microorganism Deposit Center (NPMD) under the accession numbers NITE BP-04100 and 04101. Vaginal *Lactobacillus* was cultured in Lactobacilli MRS Broth liquid medium (Becton Dickinson Difco, Franklin Lakes, NJ, USA) at 37°C under 5% CO_2_ for 24 h. Agar (Nacalai Tesque, Kyoto, Japan) was added to each liquid medium at a final concentration of 1.5% to prepare plate agar. MRS agar plates were incubated anaerobically in an anaeropack (Mitsubishi Gas Chemical Co., Inc., Tokyo, Japan) at 37°C for 24 h. The colonies were counted, and viable counts were expressed as colony-forming units per milliliter (CFU/mL).

### Cell Culture Conditions

VK2/E6E7 (ATCC CRL-2616), primary immortalized human vaginal epithelial cells, were obtained from the American Type Culture Collection (ATCC, Manassas, VA, USA). A2EN, primary immortalized human cervical epithelial cells, were obtained from Applied Biological Materials Inc. (Richmond, BC, Canada). Human endometrial epithelial cells HEC-1A (JCRB1117) were obtained from the National Institute of Biomedical Innovation, Health and Nutrition (Ibaraki, Osaka, Japan). VK2/E6E7 cells were cultured in equal parts of Keratinocyte-SFM supplemented with 50 µg/mL bovine pituitary extract (BPE), 5 ng/mL human recombinant epidermal growth factor (EGF, Thermo Fisher Scientific, Waltham, MA, USA), and 909.1 µg/mL CaCl_2_, alongside EpiLife medium (Thermo Fisher Scientific) supplemented with 909.1 µg/mL CaCl_2_. A2EN cells were cultured in a mixture of Keratinocyte-SFM supplemented with 50 µg/mL BPE, 5 ng/mL human recombinant EGF, and 909.1 µg/mL CaCl_2_. HEC-1A cells were cultured in McCoy's 5A medium (Thermo Fisher Scientific) supplemented with 10% Fetal Bovine Serum (FBS, Thermo Fisher Scientific) and 2 mM L-glutamine (Nacalai Tesque). Primocin (Invivogen, San Diego, CA, USA) was added to VK2/E6E7 or A2EN cell medium to reach a final concentration of 100 µg/mL. Penicillin–streptomycin solution (Nacalai Tesque) was added to HEC-1A cell medium to achieve a final concentration of 100 U/mL and 100 µg/mL. The use of these established, commercially available cell lines is classified by our institution as not requiring separate ethics committee approval, as they are non-identifiable and their research use is covered by the original terms of consent and acquisition. The cell lines were confirmed to be free of mycoplasma contamination. All experiments were conducted in accordance with institutional guidelines and regulatory standards.

Cells were cultured to confluence in cell culture medium at 37°C under 5% CO_2_, washed with Dulbecco's phosphate-buffered saline (DPBS), and incubated with 0.25% trypsin/0.1% ethylenediaminetetraacetic acid (EDTA) (Trypsin-EDTA, Corning, Corning, NY, USA) at 37°C under 5% CO_2_ for 5 min. After confirming cell detachment from the flask bottom, a neutralizing medium (RPMI-1640 with 10% FBS (Sigma-Aldrich Co., St. Louis, MO, USA)) was added and mixed. The cell suspension was transferred to a 15 mL tube and centrifuged (805 *×g*, 20°C, 5 min). Cells were resuspended in cell culture medium for future experiments or passaging.

### Preparation of Heat-Killed Bacterial Cells

After cultivating the bacteria, an equal volume of live bacterial culture was heat-killed at 65°C for 4 h in a water bath. After centrifugation, the bacterial cells were washed twice and resuspended in an appropriate volume of DPBS. The bacterial suspension was checked each time to ensure no live cells remained after spreading on an agar plate.

### Infection of Vaginal *Lactobacillus* in Epithelial Cells

Cells were seeded at 2.0 × 10^5^ cells/mL in 24-well plates at 37°C under 5% CO_2_ for 24 h, then washed with DPBS and dispensed into 900 µL of antibiotic-free cell growth medium. Lactobacilli at a multiplicity of infection (MOI) of 50 CFU/cell in 100 µL of DPBS were added. The plates were incubated for 4, 6, or 24 h at 37°C under 5% CO_2_.

### Number of Adherent Bacterial Cells to Vaginal Epithelial Cells

Bacterial cell counts were determined microscopically using the 4',6-diamidino-2-phenylindole (DAPI) staining method, as described previously [32]. Briefly, after incubation for 4 h with vaginal *L. crispatus*, cells were washed twice with DPBS and fixed in a 4% paraformaldehyde phosphate buffer solution (prepared by dissolving 4% paraformaldehyde powder (Nacalai Tesque) in 3 × DPBS pH 7.4) at 4°C for 18 h. After fixation, cells were washed twice with DPBS, dried, treated with 95% ethanol for 10 min, and subsequently dried again. Samples were stained with VECTASHIELD Mounting Medium containing DAPI (Vector Laboratories, Inc., Newark, CA, USA). Fluorescent images were observed and acquired via confocal microscopy (LSM980, Carl Zeiss, Oberkochen, Germany). Images were analyzed using ImageJ [33]. Microscopic counts were conducted from each of the nine images, divided into nine parts per sample, with samples blinded.

### RNA Extraction, cDNA Preparation, and Quantitative Polymerase Chain Reaction (qPCR)

After co-incubation with vaginal *L. crispatus* and epithelial cells for 6 h, the cells were washed twice with DPBS, and RNA was extracted using a FastGene RNA Premium Kit (Nippon Genetics Co., Ltd., Tokyo, Japan) according to the manufacturer's specifications. Complementary DNA (cDNA) was synthesized via reverse transcription (total 20 µL, 25°C for 5 min, 46°C for 20 min, 95°C for 1 min) using 1 µg of RNA as a template and iScript Reverse Transcription Supermix for RT-PCR (Bio-Rad Laboratories, Hercules, CA, USA). qPCR was performed using 2.5 μL of the prepared cDNA as the template with iTaq Universal SYBR Green Supermix (Bio-Rad) and the primers listed in Supplementary Table 1.

### Enzyme-Linked Immunosorbent Assay (ELISA)

HβD-2 in cell supernatant after co-incubation with *L. crispatus* for 24 h was measured using an ELISA kit (Phoenix Pharmaceuticals, Inc., Burlingame, CA, USA). Briefly, 96-well microtiter ELISA plates were washed five times with wash buffer (DPBS containing 0.05% Tween 20). Thereafter, standards and samples (100 μL/well) were added, and plates were incubated for 2 h at 20–25°C on a plate shaker (Digital Tube Rocker, Thermo Fisher Scientific). After washing the plates four times, 100 μL of biotinylated detection antibody was added per well. Plates were incubated for 2 h at room temperature on a plate shaker. After the plates were thoroughly washed, Avidin-horseradish peroxidase (HRP) was added, and the plates were incubated for 30 min at room temperature on a plate shaker. After further washing, 100 μL of 3,3′,5,5′-tetramethylbenzidine solution was added to each well. Following color development for 10–20 min at room temperature, absorbance at 450 nm was determined using a microtiter plate reader (SpectraMax iD3, Molecular Devices, LLC, San Jose, CA, USA). Extrapolation of values for sample data from standard curves was performed using SoftMax Pro (Molecular Devices, LLC).

### Statistical Analyses

Statistical analyses were performed using GraphPad Prism version 10.2.3 (GraphPad Software Inc., San Diego, CA, USA). To assess differences or correlations between the groups, we performed either the D’Agostino–Pearson omnibus normality test (number of samples; n ≥ 8) or the Shapiro–Wilk normality test (n < 8) to determine whether to use parametric or nonparametric statistics. We conducted an unpaired Student's *t*-test or a one-way analysis of variance with Tukey's multiple-comparison test for parametric data, and Spearman's correlation for nonparametric data.

## RESULTS

### Strain-Specific Induction of *HβD2* via Vaginal *L. crispatus* in Epithelial Cells

**Figure 1.**
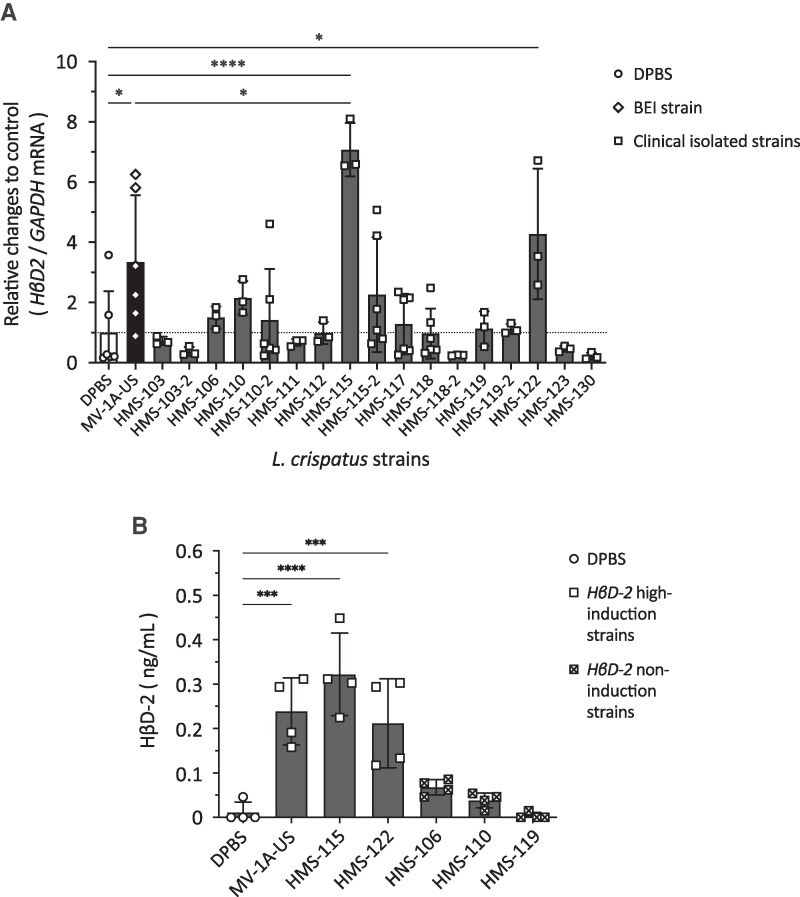
Vaginal *Lactobacillus crispatus* strains differed in their ability to enhance human β-defensin-2 (HβD-2) mRNA expression and production. *L. crispatus* was incubated with VK2/E6E7 cells at a multiplicity of infection (MOI) of 50 for (*A*) 6 or (*B*) 24 h. *A*, After RNA extraction from VK2/E6E7 cells, cDNA was prepared, and quantitative PCR was used to measure *HβD2* or *GAPDH* mRNA levels. Each value was normalized to *GAPDH* mRNA levels. Normalized value in Dulbecco's phosphate-buffered saline (DPBS)-treated cells was set to 1. Vertical axis indicates relative changes in *HβD2* mRNA compared with the DPBS control, while the horizontal axis represents the different *L. crispatus* strains. *B*, HβD-2 concentration in cell supernatant was quantified by ELISA. Vertical bars represent mean ± standard deviations. Statistically significant differences (*; *P* < .05, ***; *P* < .001, ****; *P* < .0001) are presented based on one-way analysis of variance followed by Tukey's multiple comparison test (A: DPBS vs MV-1A-US; *P* = .0315, DPBS vs HMS-115; *P* < .0001, DPBS vs HMS-122; *P* = .0498, MV-1A-US vs HMS-115; *P* = .0114, B: DPBS vs MV-1A-US; *P* = .0002, DPBS vs HMS-115; *P* < .0001, DPBS vs HMS-122; *P* = .0007). All experiments were conducted in at least three independent trials.

Significant differences were observed in the ability to enhance *HβD2* mRNA expression among the tested *L. crispatus* strains in vaginal epithelial cells (Figure 1*A*). The *HβD2* mRNA expression in the *L. crispatus* HMS-115 or HMS-122-treated groups was significantly higher than that in the DPBS-treated group (Figure 1*A*). Notably, *L. crispatus* HMS-115 showed greater *HβD2*-inducing activity than *L. crispatus* MV-1A-US, the positive control, which has a high capacity for *HβD2* mRNA induction (Figure 1*A*, Supplementary Table 2). The eighteen remaining strains tested showed no significant differences compared with the DPBS-treated group (Figure 1*A*, Supplementary Table 2). Co-incubation at MOIs of 50 and 200 with *L. crispatus* HMS-115 showed greater *HβD2*-inducing activity than at an MOI of 12.5, but no significant differences were observed in *HβD2* non-induction strains (HMS-106 and HMS-110; Supplementary Figure 1). Co-incubation for 6, 9, and 12 h with *L. crispatus* HMS-115 showed greater *HβD2*-inducing activity than that for 3 h, but no significant differences were observed in *HβD2* non-induction strains (HMS-106 and HMS-110; Supplementary Figure 2). ELISA analysis showed that the HβD-2 protein concentration in the cell supernatant was significantly higher in the groups treated with the *HβD2* mRNA high-induction strains (HMS-115 or HMS-122) than in the DPBS-treated control group or the groups treated with the *HβD2* non-induction strains, such as HMS-106, HMS-110, or HMS-119 (Figure 1*B*).

### Adhesion of *L. crispatus* Strains to Vaginal Epithelial Cells Correlated With *HβD2* Induction

**Figure 2.**
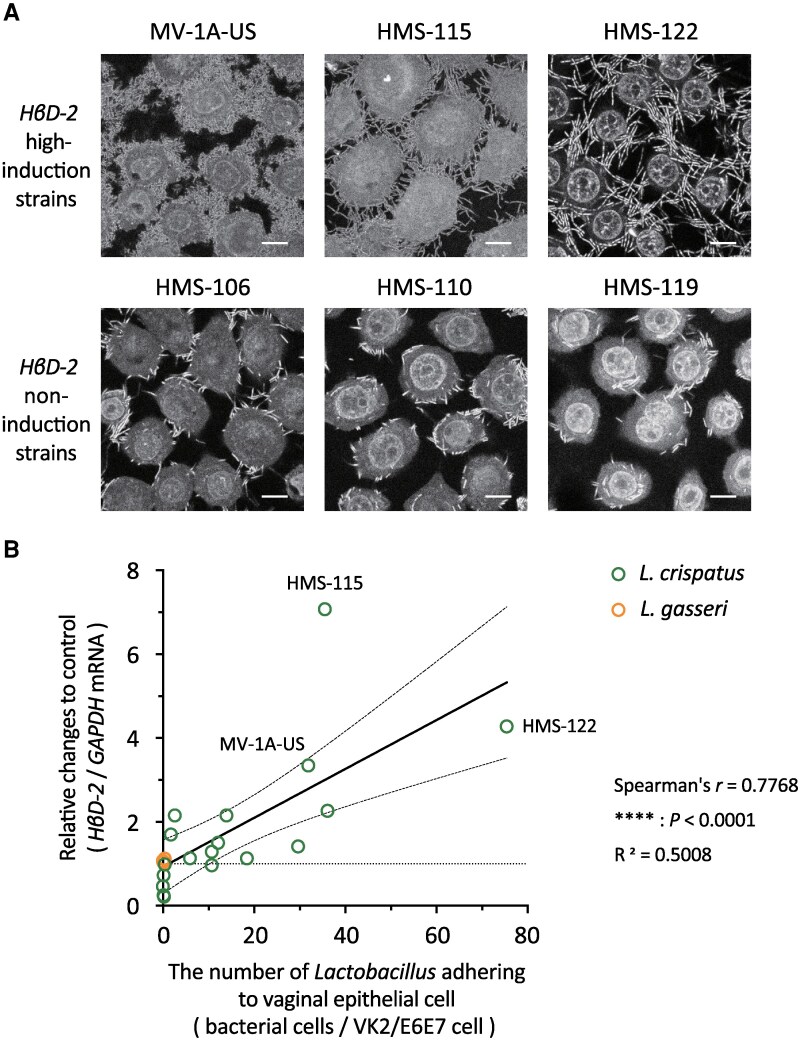
Ability of *Lactobacillus crispatus* and *Lactobacillus gasseri* to enhance human β-defensin-2 (*HβD2*) mRNA expression was associated with its binding capacity to vaginal epithelial cells. *L. crispatus* or *L. gasseri* was incubated with VK2/E6E7 at a multiplicity of infection (MOI) of 50 for (*A*) 4 or (*B*) 6 h. *A*, Nuclei of both bacterial and host cells were stained blue with DAPI. White scale bar represents 10 μm. Microscopic bacterial cell counts were performed on nine images per sample. *B*, A correlation is highlighted between induced *HβD2* mRNA expression and the number of *L. crispatus* strains that adhere to vaginal epithelial cells. Dotted lines show the 95% confidence bands of the best-fit lines. All experiments were conducted in at least three independent trials. Each dot indicates the mean values. The correlation was evaluated using Spearman's correlation coefficient; **** indicates statistically significant differences (*P* < .0001).

Strains with higher activity in enhancing *HβD2* mRNA expression (HMS-115 or HMS-122) showed greater adhesion to VK2/E6E7 cells than did strains without such activity (HMS-106, HMS-110, or HMS-119; Figure 2*A*). The microscopic counts indicated that the number of vaginal *L. crispatus* and *L. gasseri* cells adhering to VK2/E6E7 significantly correlated with their ability to enhance *HβD2* mRNA expression (Figure 2*B*). Although almost all dots fell within the 95% confidence bands of the best-fit lines, HMS-115 was far from these lines (Figure 2*B*). CBB staining and nano LC-MS/MS analysis of whole-cell SDS protein extracts from *L. crispatus* HM-638 identified the primary protein as the SLP (Supplementary Table 3, Supplementary Figure 3A). SLP size appeared to vary slightly among strains (Supplementary Figure 3B).

### Differences in Adhesive Properties and Stimulation of HβD-2 Secretion in Live and Heat-Killed HMS-115 Cells in Vaginal Epithelial Cells

**Figure 3.**
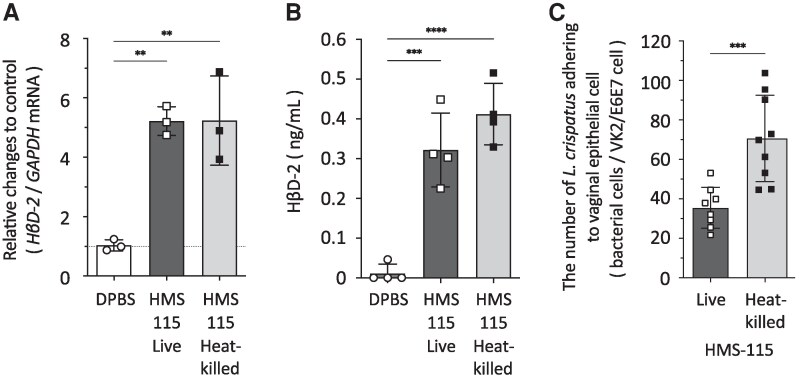
No significant differences were observed in human β-defensin-2 (*HβD*2) mRNA expression and production between live and heat-killed bacterial cells, but heat-killed cells showed higher adhesion abilities than live cells. Live or heat-killed *L. crispatus* was incubated with VK2/E6E7 cells at a multiplicity of infection (MOI) of 50 for (*A*) 6, (*B*) 24, or (*C*) 4 h. *A*, After RNA extraction from VK2/E6E7 cells, cDNA was prepared, and quantitative PCR was conducted to measure mRNA levels of HβD2 or *GAPDH*. Each value was normalized to GAPDH mRNA levels. Normalized value in Dulbecco's phosphate-buffered saline (DPBS)-treated cells was set to 1. Vertical axis indicates the relative changes compared with the DPBS control of *HβD2* mRNA, while the horizontal axis represents the live or heat-killed *L. crispatus* HMS-115. *B*, The amount of HβD-2 in the cell supernatant was measured by ELISA. *C*, Microscopic bacterial cell counts were conducted on nine images per sample. Vertical bars represent mean ± standard deviations. Statistically significant differences (**; *P* < .01, ***; *P* < .001, ****; *P* < .0001) are presented based on unpaired *t*-test or one-way analysis of variance followed by Tukey's multiple comparison test (A: DPBS vs HMS-115 Live; *P* = .0034, DPBS vs HMS-115 Heat-killed; *P* = .0033, B: DPBS vs HMS-115 Live; *P* = .0003, DPBS vs HMS-115 Heat-killed; *P* < .0001, C: Live vs Heat-killed; *P* = .0009). The experiment was conducted in at least three independent trials.

*HβD2* mRNA expression and protein concentration in the heat-killed *L. crispatus* HMS-115-treated groups were significantly higher than those in the DPBS-treated control group (Figure 3*A*, *B*). No significant difference in the ability to stimulate HβD-2 mRNA expression and protein production in vaginal epithelial cells was observed between the live and heat-killed *L. crispatus* HMS-115 cell preparations (Figure 3*A*, *B*). Conversely, adherence of heat-killed *L. crispatus* to VK2/E6E7 cells was significantly higher than that of live HMS-115 (Figure 3*C*).

### Tissue-Specific Induction of *HβD2* in Heat-Killed *L. crispatus* HMS-115 in Cervical, Endometrial, and Other Epithelial Cells

**Figure 4.**
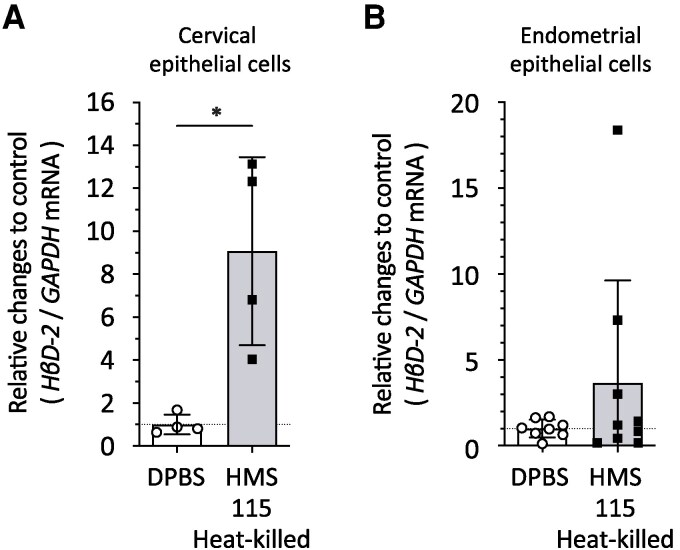
*Lactobacillus crispatus*-induced human β-defensin-2 (*HβD2*) mRNA expression in cervical epithelial cells, but not in endometrial epithelial cells. Heat-killed *L. crispatus* was incubated with (*A*) A2EN and (*B*) HEC-1A cells at a multiplicity of infection (MOI) of 50 for 6 h. After extracting RNA, cDNA was prepared, and quantitative PCR was performed to measure the mRNA levels for *HβD2* or *GAPDH*. Each value was normalized to *GAPDH* mRNA levels. Normalized value in Dulbecco's phosphate-buffered saline (DPBS)-treated cells was set to 1. Vertical axis indicates the relative changes compared with the DPBS control of *HβD2* mRNA. All experiments were conducted in at least four independent trials. Vertical bars represent mean ± standard deviations. Statistically significant difference (*; *P* < .05) is presented based on unpaired *t*-test results (A: DPBS vs HMS-115 Heat-killed; *P* = .0105).

*HβD2* mRNA expression in heat-killed *L. crispatus* HMS-115-treated groups in A2EN cells was significantly higher than that in the DPBS-treated group (Figure 4*A*). No significant differences were observed in the ability to stimulate *HβD2* mRNA expression in HEC-1A between *L. crispatus*-treated groups and the DPBS-treated group (Figure 4*B*). *HβD2* mRNA expression in heat-killed *L. crispatus* HMS-115-treated groups was significantly increased in Detroit 562 (pharyngeal) and HCT116 (colorectal) cell lines than in the DPBS-treated group (Supplementary Figure 4).

### Differential Modulation of Inflammatory and Mucin Expression in Live and Heat-Killed *L. crispatus* in Vaginal Epithelial Cells

**Figure 5.**
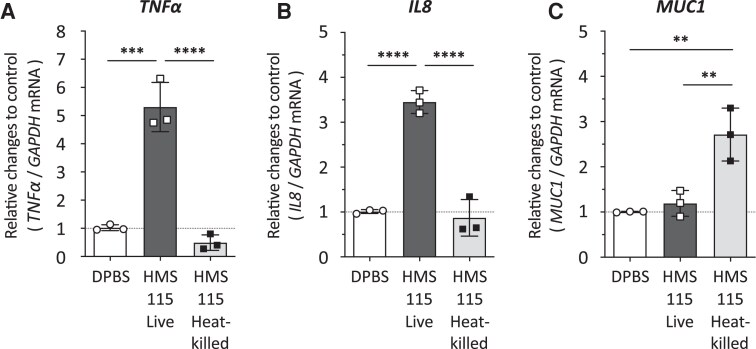
Impact of *Lactobacillus crispatus* bacterial viability on vaginal epithelial cells. *L. crispatus* was incubated with VK2/E6E7 cells at a multiplicity of infection (MOI) of 50 for 6 h. After extracting RNA from VK2/E6E7 cells, cDNA was prepared, and quantitative PCR was performed to measure mRNA levels for *TNFα* (A), *IL8* (B), and *MUC1* (C). Each value was normalized to *GAPDH* mRNA levels, with the value in Dulbecco's phosphate-buffered saline (DPBS)-treated cells set to 1. Vertical axis represents the relative changes for each target mRNA compared to the DPBS control, while the horizontal axis shows the live or heat-killed *L. crispatus* HMS-115. All experiments were conducted in three independent trials. Vertical bars represent mean ± standard deviations. Statistically significant differences (**; *P* < 0.01, ***; *P* < 0.001, ****; *P* < 0.0001) are presented based on one-way analysis of variance followed by Tukey's multiple comparison test (A: DPBS vs HMS-115 Live; *P* = 0.0002, HMS-115 Live vs Heat-killed; *P* < 0.0001, B: DPBS vs HMS-115 Live; *P* < 0.0001, HMS-115 Live vs Heat-killed; *P* < 0.0001, C: DPBS vs HMS-115 Heat-killed; *P* = 0.0034, HMS-115 Live vs Heat-killed; *P* = 0.0061).

Live *L. crispatus* HMS-115 cells enhanced the expression of pro-inflammatory cytokines such as *IL8* and *TNFα* in VK2/E6E7 cells, whereas heat-killed cells did not (Figure 5*A*, *B*). In contrast, *MUC1* mRNA expression significantly increased upon heat-killed *L. crispatus* HMS-115 treatment compared to live-cell treatment (Figure 5*C*).

## DISCUSSION

**Figure 6.**
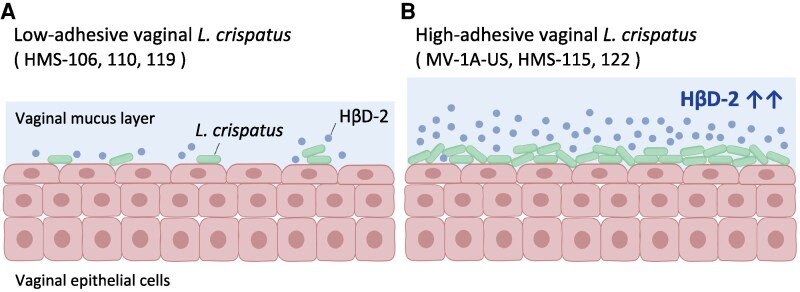
Schematic representation of the interaction between vaginal *Lactobacillus crispatus* and human β-defensin-2 (HβD-2) production. HβD-2 mRNA expression and production in vaginal epithelial cells varied significantly across *L. crispatus* strains. Vaginal *L. crispatus* with strong adhesion capability increased HβD-2 production in vaginal epithelial cells.

This study examined seventeen *L. crispatus* and three *L. gasseri* strains isolated from the vaginas of healthy Japanese women for their ability to enhance HβD-2 production in vaginal epithelial cells. To our knowledge, this is the first report to show evident variation in *HβD2* mRNA-enhancing activity among vaginal *L. crispatus* isolates, statistically linked to the number of *L. crispatus* cells that adhere to vaginal epithelial cells. Among the tested strains, HMS-115 exhibited the highest activity, which persisted even after heat-killing bacterial cells at 65°C for 4 h. In contrast, live HMS-115 increased pro-inflammatory cytokine mRNA expression (*IL8* and *TNFα*) in vaginal epithelial cells, whereas heat-killed HMS-115 did not. However, heat-killed *L. crispatus* enhanced *MUC1* mRNA expression, whereas live *L. crispatus* did not. Overall, selected *L. crispatus* strains, such as HMS-115, which strongly adhere to vaginal cells and stimulate HβD-2 production (Figure 6), may help address female reproductive conditions, including sexually transmitted diseases and PTB, without triggering an excessive inflammatory response.

Constitutive *HβD2* mRNA expression has been observed in the foreskin, lung, and trachea; however, it is significantly lowered in the kidney and salivary glands [21]. This indicates that *HβD2* mRNA expression varies among different organs and tissues. *HβD2* mRNA expression increased upon heat-killed bacteria (*P. aeruginosa*, *E. coli*, *S. epidermidis*, *S. aureus*, and *C. albicans*) exposure in primary human foreskin keratinocytes [21]. Dinulos et al. showed that exposure to *P. aeruginosa*, *E. coli*, *S. epidermidis*, and *S. aureus* induced *HβD2* mRNA expression in human neonatal foreskin [34]. However, *Streptococcus pyogenes* strains were weak and inconsistent inducers of *HβD2* [34], suggesting that bacterial contact with human foreskin can promote *HβD2* mRNA expression, depending on the strain. In this study, *L. crispatus* strains with higher HβD-2 mRNA expression and protein production exhibited greater adhesive ability toward vaginal cells, whereas strains lacking *HβD2* mRNA expression did not. This suggested that effective interaction between *L. crispatus* and vaginal epithelial cells is essential for increased *HβD2* mRNA expression. Additionally, this enhancement was detected in cervical epithelial cells, but not in endometrial epithelial cells. Rizzo et al. reported that eye-isolated *L. crispatus* increased HβD-2 levels in HeLa cells, a human cervical cancer cell line [29]. In contrast, Harder et al. found that an increase in *HβD2* mRNA expression was not observed in the uterus [21]. These findings align with our results, demonstrating tissue- and cell-specific *L. crispatus* activation.

Schlee et al. demonstrated that *L. acidophilus* and *L. fermentum* dose-dependently induced *HβD2* mRNA expression in Caco-2 cells [28]. Recently, a cohort study of 2000 pregnant women found that high vaginal HβD-2 levels were associated with a lower risk of PTB [27]. Furthermore, low HβD-2 levels increased PTB risk, even in the presence of a *Lactobacillus*-dominant cervicovaginal microbiota, indicating that HβD-2 levels are independent of dominant bacterial species [27]. Our findings suggested that *L. crispatus*-induced HβD-2 production by vaginal epithelial cells varies significantly by bacterial strains. Additionally, the significant positive correlation between HβD-2 secretion stimulation by clinically isolated *L. crispatus* and its adherence to vaginal epithelial cells suggested that bacterial cell numbers may influence HβD-2 production. Viable bacterial counts of *L. crispatus*-dominated samples in healthy Japanese women varied more than 70-fold [31]. Therefore, both the bacterial load of indigenous *L. crispatus* and their adhesive ability toward vaginal epithelial cells likely influence HβD-2 production.

*L.*  *crispatus* retained its ability to adhere to vaginal epithelial cells and promote HβD-2 production even after being heat-killed at 65°C for 4 h. The capacity to induce *HβD2* mRNA expression was also observed in various epithelial cell types, including cervical, pharyngeal, and colorectal epithelial cells, but not in endometrial cells. These results indicate that identical bacterial component(s) trigger *HβD2* mRNA expression across responsive epithelial cell types. Lactobacilli adhesion involves (1) SLPs, (2) cell wall-anchored proteins, and (3) housekeeping proteins [35]. SLPs are composed of a single protein or glycoprotein with a regular crystalline structure, accounting for approximately 20% of total proteins in the bacterial cell. As SLPs are located in the outermost bacterial cell layer [36], they perform various functions, including environmental stress adaptation [37] and host cell adhesion [38]. S-layer-protein-encoding genes have been cloned and sequenced from species such as *Levilactobacillus brevis*, *L. acidophilus*, *L. helveticus*, and *L. crispatus* [36]. Kobatake et al. reported that the SLP of *L. helveticus* SBT 2171 enhanced HβD-2 production in colorectal epithelial cells, and heated SLP induced *HβD-2* at levels comparable to those induced by intact SLP [30]. In this study, CBB staining and nano LC-MS/MS analysis of whole-cell SDS protein extracts from *L. crispatus* identified SLP as the major protein, with marginal size variation among strains. These findings align with previous studies on genetic diversity and low amino acid identity among lactobacilli SLPs [39], including strain-specific differences in *L. helveticu*s and *Lactiplantibacillus plantarum* [39, 40]. Although HMS-115 did not show high correlation between bacterial adherence and *HβD2* expression, this was suggested to reflect its well-aggregative characteristics, which are well-associated with SLPs. The correlation between the ability to enhance HβD-2 production in vaginal epithelial cells and strain-specific SLP structures in *L. crispatus* will be clarified in the future.

Although HβD-2 expression and production were unaffected by *L. crispatus* viability, pro-inflammatory cytokines, such as *IL8* and *TNFα*, were diminished upon heat-killed *L. crispatus* treatment compared with live-cell treatment. This difference may arise from live cells producing certain metabolites absent in heat-killed preparations. Delgado-Diaz et al. reported that acetate induces *TNFα* expression via TLR-agonists in vaginal epithelial cells [41]. Hydrogen peroxide, secreted by *L. crispatus* [42], induces *IL8* expression in Caco-2 cells [43]. Hearps et al. found that lactic acid, primarily produced by lactobacilli, triggers an anti-inflammatory response and inhibits pro-inflammatory mediators in human cervicovaginal epithelial cells [44]. Decout et al. demonstrated that SLPs mask TLR2 ligands in *L. crispatus*, inhibiting TLR2-dependent pro-inflammatory pathway activation [45]. Collectively, bacterial metabolites that enhance pro-inflammatory activity in vaginal epithelial cells may be altered or absent in the heat-killed preparation.

Unlike HβD-2 and pro-inflammatory markers, *MUC1* expression was significantly increased by heat-killed *L. crispatus*. MUC1 is a transmembrane glycoprotein that exhibits both adhesive and antiadhesive properties, effectively creating a barrier against invading microbes on epithelial cell surfaces [46]. MUC1 is an abundantly expressed mucin in vaginal epithelial cells [46]. Lactic acid bacteria stimulate mucin production [47, 48], and Mack et al. reported that *Lactobacillus* strain adherence to intestinal epithelial cells induces extracellular mucin secretion [47]. Therefore, *L. crispatus* adhesion to vaginal epithelial cells may induce *MUC1* expression. In this study, more heat-killed HMS-115 cells adhered to VK2/E6E7 cells than live HMS-115 cells. Some reports showed that heat-killed *Lactobacillus* cells adhered significantly more than live cells [49, 50]. Coconnier et al. suggested that heat-killed bacteria retain or even expose surface adhesion components [50]. Thus, the difference in the number of adhered live and heat-killed *L. crispatus* cells in vaginal epithelial cells may affect HMS-115's ability to induce *MUC1* mRNA expression.

However, this study has some limitations. The strain-specific key components of *L. crispatus* that enhance HβD-2 production and adhesion remain unidentified. Additionally, this research used only immortalized cell lines without immune or hormonal components. Furthermore, studies on the antimicrobial properties of the selected *L. crispatus* strains, along with subsequent clinical validation, are required.

In conclusion, the selected vaginal *L. crispatus* strains (HMS-115 and HMS-122) that stimulate HβD-2 production in vaginal epithelial cells also exhibit strong adherence to vaginal epithelial cells. HβD-2 protects against bacteria, fungi, and some viruses [26], and its production is also positively correlated with spontaneous birth [27]. Additionally, upregulated mucin has a protective effect by preventing pathogens and commensals from binding to mucosal cells. Consequently, *L. crispatus* strains that strongly enhance high HβD-2 production in vaginal epithelial cells may be candidates for preventing sexually transmitted diseases and PTB.

## Supplementary Material

ofag193_Supplementary_Data

## Supplementary Data

Supplementary materials are available at *Open Forum Infectious Diseases* online. Consisting of data provided by the authors to benefit the reader, the posted materials are not copyedited and are the sole responsibility of the authors, so questions or comments should be addressed to the corresponding author.
