# Supplementary material for: Induction of Human β-Defensin-2 by Vaginal Lactobacillus crispatus Strains in Vaginal Epithelial Cells Correlates With Their Adhesion Abilities
Source: Open Forum Infect Dis. 2026 Apr 25;13(4):ofag193. doi: 10.1093/ofid/ofag193 (PMC13089552; doi:10.1093/ofid/ofag193)
Supplement: ofag193_Supplementary_Data [file ofag193_supplementary_data.zip › ITO_Supplementary_Figures_version_3.docx]

**
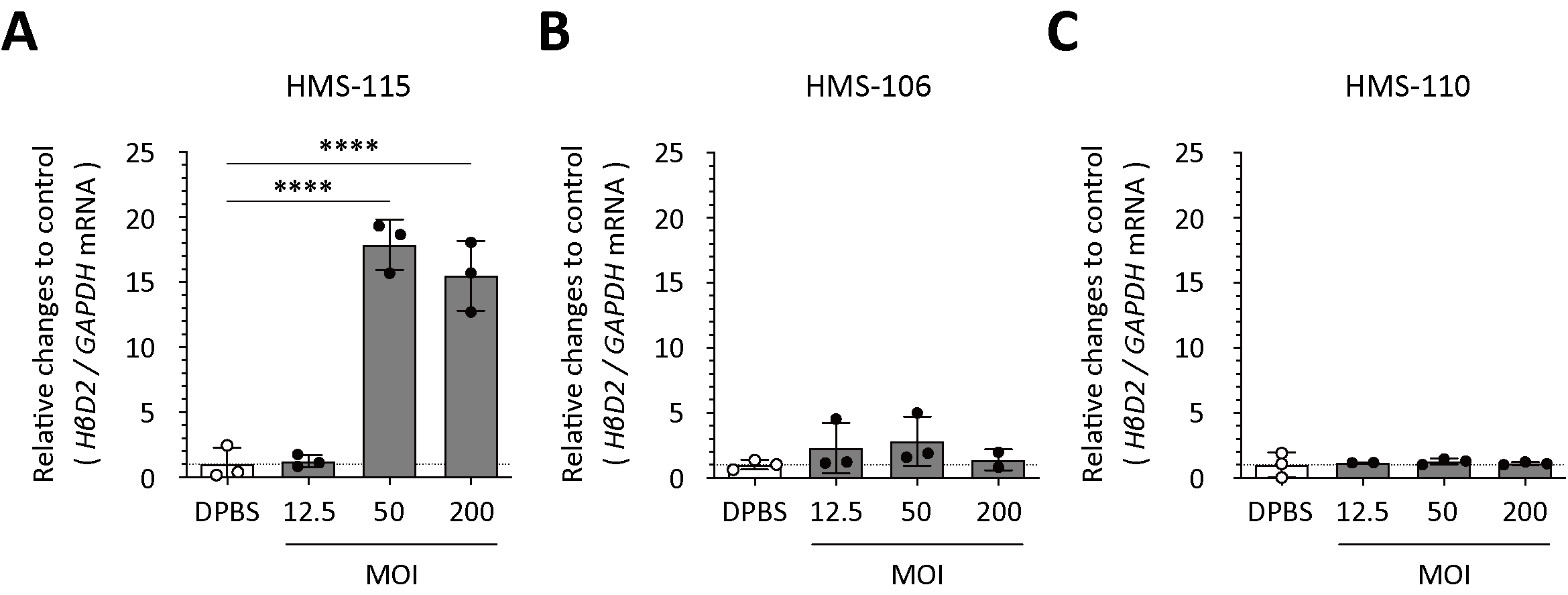
Supplementary Figures**

**Supplementary Figure 1. Human defensin-2 (*HβD2*) mRNA expression differed with the multiplicity of infection (MOI) of *L. crispatus* in vaginal epithelial cells.**

*L. crispatus* (A) HMS-115, (B) HMS-106, or (C) HMS-110 was incubated with VK2/E6E7 cells at a multiplicity of infection (MOI) of 12.5, 50, and 200 for 6 h. After RNA extraction from VK2/E6E7 cells, cDNA was prepared, and quantitative PCR was used to measure *HβD2* or *GAPDH* mRNA levels. Each value was normalized to *GAPDH* mRNA levels. Normalized value in DPBS-treated cells was set to 1. All experiments were conducted at least three times, except for HMS-106 at MOI 200 and HMS-110 at MOI 12.5. Vertical bars represent mean ± standard deviations. Statistically significant differences (****; *P* < 0.0001) are presented based on one-way ANOVA followed by Tukey's multiple comparison test (DPBS vs HMS-115 MOI 50, *P* < 0.0001; DPBS vs HMS-115 MOI 200, *P* < 0.0001).

**
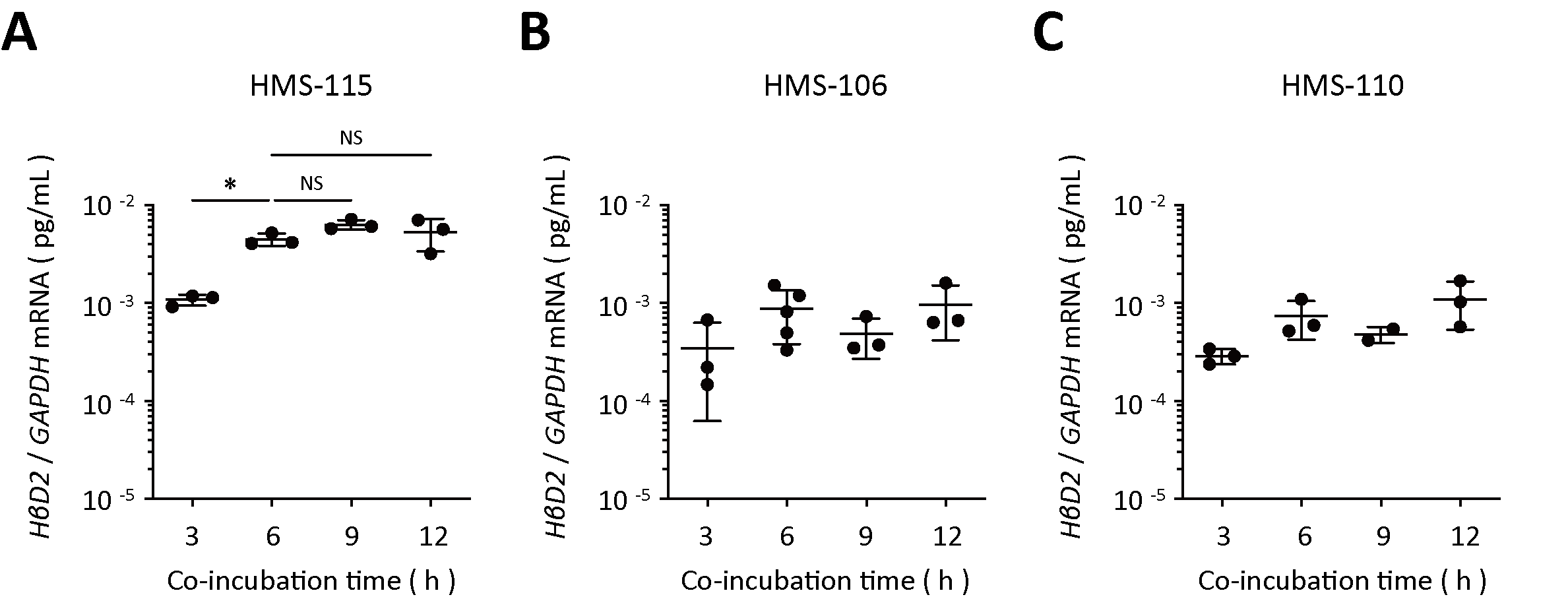
**

**Supplementary Figure 2. Human defensin-2 (*HβD2*) mRNA expression differed with the duration of *L. crispatus* infection in vaginal epithelial cells.**

*L. crispatus* (A) HMS-115, (B) HMS-106, or (C) HMS-110 was incubated with VK2/E6E7 cells at an MOI of 50 for 3, 6, 9, and 12 h. After RNA extraction from VK2/E6E7 cells, cDNA was prepared, and quantitative PCR was used to measure *HβD2* or *GAPDH* mRNA levels. Each value was normalized to *GAPDH* mRNA levels. Normalized value in DPBS-treated cells was set to 1. All experiments were conducted at least three times, except for HMS-110 at 9 h. Vertical bars represent mean ± standard deviations. Statistically significant difference (*; *P* <0.05) is presented based on one-way ANOVA followed by Tukey's multiple comparison test (3 h vs. 6 h; *P* = 0.0215). N.S. represents non-significant differences.

**Supplementary Figure 3. Coomassie blue staining of whole-cell SDS protein extracts from vaginal *L. crispatus* cells indicated that S-layer protein (SLP) size varied among strains.**

Vaginal *L. crispatus* was incubated and harvested to form a pellet. The pellet was mixed with 1/10 volume of 1 × sample buffer containing 100 mM dithiothreitol and boiled for 5 min. The supernatant was subsequently used for SDS-PAGE, and the protein bands were stained with Coomassie blue. An approximately 46 kDa protein in whole-cell SDS extracts from *L. crispatus* 125-2-CHN was identified as the SLP by nano-LC-MS/MS. An asterisk indicates that the band is considered the SLP of each strain.

**Supplementary Figure 4. *L. crispatus* induced human defensin-2 (*HβD2*) mRNA expression in pharyngeal and colorectal epithelial cells.**

Heat-killed *L. crispatus* strains were incubated with (A) Detroit 562 and (B) HCT116 cells at an MOI of 50 for 6 h. After RNA extraction, cDNA was prepared, and quantitative PCR was performed to measure mRNA levels of *HβD2* and *GAPDH*. Each value was normalized to *GAPDH* mRNA levels, with the value in DPBS-treated cells set to 1. The vertical axis shows the relative changes in *HβD2* mRNA compared to the DPBS control. All experiments were conducted in four independent trials. Vertical bars represent mean ± standard deviations. Statistically significant differences (*; *P* < 0.05, ***; *P* < 0.001) are indicated based on unpaired *t*-test results (A: DPBS vs HMS-115 Heat-killed, *P* = 0.0002; B: DPBS vs HMS-115 Heat-killed, *P* = 0.0212).
