# Supplementary material for: Induction of Human β-Defensin-2 by Vaginal Lactobacillus crispatus Strains in Vaginal Epithelial Cells Correlates With Their Adhesion Abilities
Source: Open Forum Infect Dis. 2026 Apr 25;13(4):ofag193. doi: 10.1093/ofid/ofag193 (PMC13089552; doi:10.1093/ofid/ofag193)
Supplement: ofag193_Supplementary_Data [file ofag193_supplementary_data.zip › ITO_Supplementary_Materials_version_3.docx]

**Supplementary Materials**

**Bacterial Strains and Culture Conditions**

*Lactobacillus crispatus* 125-2-CHN was obtained from BEI Resources, whereas others were from our previous study [1]. Vaginal *Lactobacillus* was cultured in Lactobacilli MRS Broth liquid medium (Becton Dickinson Difco) at 37 °C under 5% CO_2_ for 24 h.

**Cell Culture Conditions**

Detroit 562 (ATCC CCL-138), a human pharyngeal cell line, was obtained from the American Type Culture Collection (ATCC). HCT116 (RCB2979), a human colon cell line, was obtained from RIKEN BRC via the National BioResource Project of the MEXT/AMED (Tsukuba, Ibaraki, Japan). Detroit 562 cells were cultured in Eagle's minimal essential medium (Thermo Fisher Scientific) supplemented with 10% FBS, 1% non-essential amino acids, 2 mM L-glutamine, 1 mM sodium pyruvate (FUJIFILM Wako Pure Chemical Co., Inc., Osaka, Japan), and 1,500 mg/L sodium bicarbonate (FUJIFILM Wako Pure Chemical Co.). HCT116 cells were grown in McCoy's 5A medium containing 10% FBS. Penicillin-streptomycin solution (Nacalai Tesque) was added to the media for Detroit 562 or HCT116 cells to achieve final concentrations of 100 Units/mL and 100 µg/mL, respectively.

**Infection of Vaginal *Lactobacillus* in Epithelial Cells**

Cells were seeded at 2.0 × 10^5^ cells/mL in 24-well plates at 37 °C under 5% CO_2_ for 24 h, then washed with DPBS and dispensed into 900 µL of antibiotic-free cell growth medium. Lactobacilli at a multiplicity of infection (MOI) of 12.5, 50, or 200 CFU/cell in 100 µl of DPBS were added. The plates were incubated for 3, 6, 9, or 12 h at 37 °C under 5% CO_2_.

**Coomassie Blue Staining**

Vaginal *L. crispatus* was incubated and harvested to form a pellet. Thereafter, it was mixed with 1/10 volume of 1 × sample buffer (1 × SB, 62.5 mM Tris-HCl (pH 6.8), 1% SDS, 100 mM dithiothreitol, 6.25% glycerol, 0.0025% bromophenol blue), and boiled for 5 min. The supernatant was run on SuperSep Ace, 10% (FUJIFILM Wako Pure Chemical Co.) at 200 V for 30 min. Protein bands were stained with Coomassie Brilliant Blue (CBB; Rapid Stain CBB Kit, Nacalai Tesque) for 20 min, then shaken in purified water overnight to decolorize any excess CBB.

**Nano Liquid Chromatography-Tandem Mass Spectrometry (LC-MS/MS)**

After CBB staining, corresponding protein bands were excised. Nano LC-MS/MS analysis was conducted at the Japan Proteomics Mass Spectrometry Laboratory (Miyagi, Japan).

**References**

1. Ito M, Kataoka M, Sato Y, Nachi H, Nomoto K, Okada N. Diverse vaginal microbiota in healthy Japanese women: a combined relative and quantitative analyses. Front Cell Infect Microbiol **2025**; 14:1487990.

2. Rajput S, Volk-Draper LD, Ran S. TLR4 is a novel determinant of the response to paclitaxel in breast cancer. Mol Cancer Ther **2013**; 12:1676–87.

3. van Galen P, Kreso A, Wienholds E, et al. Reduced lymphoid lineage priming promotes human hematopoietic stem cell expansion. Cell Stem Cell **2014**; 14:94–106
