# Supplementary material for: Induction of Human β-Defensin-2 by Vaginal Lactobacillus crispatus Strains in Vaginal Epithelial Cells Correlates With Their Adhesion Abilities
Source: Open Forum Infect Dis. 2026 Apr 25;13(4):ofag193. doi: 10.1093/ofid/ofag193 (PMC13089552; doi:10.1093/ofid/ofag193)
Supplement: ofag193_Supplementary_Data [file ofag193_supplementary_data.zip › ITO_Supplementary_Table_version_3.docx]

**Supplementary Tables**

**Supplementary Table 1. Primers used in the study**

| Target | Primer name | Primer sequence (5'-3') | Product length (bp) | Reference |
| --- | --- | --- | --- | --- |
| *HβD2* | HBD-2 F | CCAGCCATCAGCCATGAGGG | 255 | This study |
|  | HBD-2 R | GGAGCCCTTTCTGAATCCGCA |  |  |
| *TNFα* | TNF-α F | ATGAGCACTGAAAGCATGATCC | 217 | [2] |
|  | TNF-α R | GAGGGCTGATTAGAGAGAGGTC |  |  |
| *IL8* | IL-8 F | GAAACCACCGGAAGGAACCATC | 92 | This study |
|  | IL-8 R | GAGCTGCAGAAATCAGGAAGGC |  |  |
| *MUC1* | MUC1 F | GATACCTACCATCCTATGAGCGAG | 136 | This study |
|  | MUC1 R | GGTTTGTGTAAGAGAGGCTGC |  |  |
| *GAPDH* | GAPDH F | GGTGGTCTCCTCTGACTTCAACA | 127 | [3] |
|  | GAPDH R | GTTGCTGTAGCCAAATTCGTTGT |  |  |

**Supplementary Table 2. Summary of strain characteristics**

| **Strains** | | **Growth phase**  (after 24 h incubation) | **Adhesion**^*1^  (bacterial cells /VK2/E6E7 cell) | ***HβD2* expression**^*2^  (Relative changes to control, *HβD2*/*GAPDH* mRNA) | **Significant differences in *HβD2* expression**  (*P* values to control and to *L. crispatus* MV-1A-US) |
| --- | --- | --- | --- | --- | --- |
| *L. crispatus* | MV-1A-US | Stationary (early) | 31.9 | 3.34 | * (*P* = 0.0315), - |
|  | HMS-103 | Stationary (early) | 0.7 | 1.70 | N.S., N.S. |
|  | HMS-103-2 | Stationary (early) | 0.1 | 0.73 | N.S., N.S. |
|  | HMS-106 | Stationary (early) | 12.1 | 1.50 | N.S., N.S. |
|  | HMS-110 | Stationary (early) | 13.9 | 2.15 | N.S., N.S. |
|  | HMS-110-2 | Stationary (early) | 29.6 | 1.41 | N.S., N.S. |
|  | HMS-111 | Stationary (early) | 2.6 | 2.15 | N.S., N.S. |
|  | HMS-112 | Stationary (early) | 0.3 | 0.98 | N.S., N.S. |
|  | HMS-115 | Stationary (early) | 35.5 | 7.07 | **** (*P* < 0.0001),  * (*P* = 0.0114) |
|  | HMS-115-2 | Stationary (early) | 36.1 | 2.26 | N.S., N.S. |
|  | HMS-117 | Stationary (early) | 10.7 | 1.29 | N.S., N.S. |
|  | HMS-118 | Stationary (early) | 10.6 | 0.97 | N.S., N.S. |
|  | HMS-118-2 | Stationary (early) | 0.1 | 0.25 | N.S., N.S. |
|  | HMS-119 | Stationary (early) | 5.9 | 1.13 | N.S., N.S. |
|  | HMS-119-2 | Stationary (early) | 18.4 | 1.12 | N.S., N.S. |
|  | HMS-122 | Stationary (early) | 75.4 | 4.28 | * (*P* = 0.0498), N.S. |
|  | HMS-123 | Stationary (early) | 0.0 | 0.46 | N.S., N.S. |
|  | HMS-130 | Stationary (early) | 0.2 | 0.22 | N.S., N.S. |
| *L. gasseri* | HMS-103 | Stationary (early) | 0.0 | 1.07 | N.S., - |
|  | HMS-103-2 | Stationary (early) | 0.0 | 1.04 | N.S., - |
|  | HMS-103-3 | Stationary (early) | 0.0 | 1.04 | N.S., - |

*L. crispatus* was incubated with VK2/E6E7 cells at a multiplicity of infection (MOI) of 50 for 4^*1^ or 6 h^*2^. ^*1^ Nuclei of both bacterial and host cells were stained blue with DAPI. White scale bar represents 10 μm. Microscopic bacterial cell counts were performed on nine images per sample. ^*2^ After RNA extraction from VK2/E6E7 cells, cDNA was prepared, and quantitative PCR was used to measure *HβD2* or *GAPDH* mRNA levels. Each value was normalized to *GAPDH* mRNA levels. Relative changes in *HβD2* mRNA compared to the DPBS control. Statistically significant differences (*; *P* < 0.05, ***; *P* < 0.001, ****; *P* < 0.0001) are presented based on one-way analysis of variance followed by Tukey's multiple comparison test (A: DPBS vs MV-1A-US; *P* = 0.0315, DPBS vs HMS-115; *P* < 0.0001, DPBS vs HMS-122; *P* = 0.0498, MV-1A-US vs HMS-115; *P* = 0.0114, B: DPBS vs MV-1A-US; *P* = 0.0002, DPBS vs HMS-115; *P* < 0.0001, DPBS vs HMS-122; *P* = 0.0007). Experiments were conducted in at least three independent trials. N.S. represents non-significant differences.

**Supplementary Table 3. Mascot search results for the *L. crispatus* protein**

| Identified Protein | | Predicted Mol. Mass (Da) | Score | Peptide | Sequence Coverage (%) | Accession in Mascot Search Result |
| --- | --- | --- | --- | --- | --- | --- |
| **1** | **S-layer protein** | **46,947** | **557** | **43** | **40** | **PKZ84372.1** |
| 2 | SLAP domain-containing protein | 44,382 | 138 | 7 | 16 | WP_230456275.1 |
